# New insights in the molecular events underlying actinorhizal nodulation in the tropical tree *Casuarina glauca*

**DOI:** 10.1186/1753-6561-5-S7-O33

**Published:** 2011-09-13

**Authors:** Claudine Franche, Hassen Gherbi, Meriem Benabdoun, Sergio Svistoonoff, Valerie Hocher, Didier Bogusz

**Affiliations:** 1 Rhizogenese, Institut de Recherche pour le Développement, 911 avenue Agropolis, BP 64501, 34394 Montpellier Cedex 5, France; 2 Laboratoire de Génétique, Biochimie et Biotechnologies Végétales, Département de Biologie et d'Ecologie, Université Mentouri, Route Ain El Bey, Constantine, Algérie

## Background

Actinorhizal root nodules result from the interaction between a nitrogen-fixing actinomycete called *Frankia* and roots of dicotyledonous trees and shrubs belonging to 8 plant families and 25 genera. Most actinorhizal plants are capable of high rates of nitrogen fixation comparable to those found in legumes. As a consequence, these trees are able to grow in poor and disturbed soils and are important elements in plant communities worldwide. This facility for adaptation has drawn great interest to actinorhizal plants, particularly to several species of *Casuarinaceae* such as *Casuarina glauca*, which can be used for fuelwood production, agroforestry, and land reclamation in the tropics and subtropics.

The basic knowledge of the symbiotic association between *Frankia *and actinorhizal plants is still poorly understood, although it offers striking differences with the *Rhizobium*-legume symbiosis [1]. Recently, the development of genomics in some actinorhizal plants such as *C. glauca*[2], together with the possibility to obtain transgenic actinorhizal plants following *Agrobacterium* gene transfer [3], offer new approaches to understand the molecular basis of the actinorhizal process. We will highlight recent progress in the molecular knowledge of the early stages of the actinorhizal symbiosis. A comparative analysis of the symbiotic pathway in actinorhizal trees and legumes will be presented.

## Methods

*Plant material. Casuarina glauca*Sieb. Ex Spreng seeds were obtained from B&T World Seeds (Aigues-Vives, France) and germinated in sterile conditions as described [4].

*Genetic transformation of* Casuarina glauca. Composite plants of *C. glauca*were obtained following the genetic transformation by *Agrobacterium rhizogenes* A4RS containing the appropriate binary vector [5]. For promoter analyses, binary vectors were derived from pBIN19 and for RNAi experiments, hairpin constructs were cloned into the pHKN29 vector using standard molecular methods.

*Nodulation by Frankia.* Composite and control plants were nodulated with a suspension of *Frankia*CcI3 [4].

## Results and discussion

### CgSymRK is involved in the actinorhizal signal transduction pathway

The development of genetic and genomic tools for the model legumes *M. truncatula*a nd *Lotus japonicus *has greatly facilitated the cloning of genes required for root symbiosis [6]. Some of these genes were found to be involved in the establishment of both rhizobia and mycorrhiza symbioses, and designated as common SYM genes constituting the common SYM pathway. They include genes encoding a leucine-rich-repeat (LRR) receptor kinase (SymRK), cation channels, nuclear pore complex proteins, a calcium and calmodulin-dependent protein kinase (CCaMK) and a nuclear coiled protein. The question was raised whether some of these symbiotic genes were shared in the signal transduction pathway in response to *Frankia* factors and rhizobial Nod factors.

To answer this question, a functional study of *CgSymRK*, a gene isolated from *C. glauca*, homologous to the receptor-like kinase gene *SymRK* required for nodulation and mycorrhization in legumes, was undertaken. Downregulation of *CgSymRK* resulting from a RNA interference approach revealed that the frequency of nodulated RNAi-*CgSymRK *plants was reduced 2-fold compared to control *C. glauca *plants [7]. In addition, a range of morphological alterations was observed in the down-regulated CgSymRK-nodules. Additional experiments revealed that CgSymRK was also necessary for the establishment of the symbiosis with the arbuscular mycorrhiza *Glomus intraradices*. Therefore, the function of SymRK is conserved between legumes and actinorhizal plants.

### Search for other candidates of the signalling pathway

Our group has developed the first genomic platform to identify plant genes involved in the symbiotic process between *Frankia *and *C. glauca *[2]. Based on the comparison with legumes sequences, ESTs sharing significant homologies with genes from the Nod signalling pathway were identified. Functional analyses of the candidate orthologues to the *CCaMK *and *NIN* genes are in progress. CCaMK is a calcium and calmodulin-dependent protein kinase that is presumed to decode and transduce Nod-factor specific calcium spiking response in legumes. Preliminary experiments suggest that CgCCaMK is necessary for both nodulation and endomycorrhization in *C. glauca*, thereby suggesting a conservation of the whole common SYM pathway in the actinorhizal plants.

## Conclusion

Although several actinorhizal genera contain species that are economically important in forestry and land regeneration, progress has been slower in our knowledge of the actinorhizal symbiosis than that of the legume rhizobium interaction. The availability of a method for the genetic transformation of Casuarinas has resulted in a major breakthrough since it opened the way to functional gene analysis in the actinorhizal host plant. Deep sequencing analyses are now in progress in *C. glauca *and mining these data will undoubtedly to contribute to dissecting the molecular dialogue between *Frankia *and the host plant in the different stages of development of the actinorhizal nodule.
